# In silico discovery of potential PPI inhibitors for anti-lung cancer activity by targeting the CCND1-CDK4 complex via the P21 inhibition mechanism

**DOI:** 10.3389/fchem.2024.1404573

**Published:** 2024-06-18

**Authors:** Weijian Tang, Tao Shen, Zhoumiao Chen

**Affiliations:** Department of Thoracic Surgery, Sir Run Run Shaw Hospital, Zhejiang University School of Medicine, Hangzhou, China

**Keywords:** CCND1-CDK4 inhibition, pharmacophore matching, molecular dynamics simulation, NSCLC, protein interaction (PPI)

## Abstract

Non-Small Cell Lung Cancer (NSCLC) is a prevalent and deadly form of lung cancer worldwide with a low 5-year survival rate. Current treatments have limitations, particularly for advanced-stage patients. P21, a protein that inhibits the CCND1-CDK4 complex, plays a crucial role in cell proliferation. Computer-Aided Drug Design (CADD) based on pharmacophores can screen and design PPI inhibitors targeting the CCND1-CDK4 complex. By analyzing known inhibitors, key pharmacophores are identified, and computational methods are used to screen potential PPI inhibitors. Molecular docking, pharmacophore matching, and structure-activity relationship studies optimize the inhibitors. This approach accelerates the discovery of CCND1-CDK4 PPI inhibitors for NSCLC treatment. Molecular dynamics simulations of CCND1-CDK4-P21 and CCND1-CDK4 complexes showed stable behavior, comprehensive sampling, and P21’s impact on complex stability and hydrogen bond formation. A pharmacophore model facilitated virtual screening, identifying compounds with favorable binding affinities. Further simulations confirmed the stability and interactions of selected compounds, including 513457. This study demonstrates the potential of CADD in optimizing PPI inhibitors targeting the CCND1-CDK4 complex for NSCLC treatment. Extended simulations and experimental validations are necessary to assess their efficacy and safety.

## 1 Introduction

Non-small cell lung cancer (NSCLC) is the most common type of lung cancer, accounting for approximately 85% of all lung cancer cases (Niu et al., 2023). It is a highly aggressive and lethal malignancy with limited treatment options, highlighting the urgent need for innovative therapeutic approaches (Li et al., 2023). One promising avenue for targeted therapy in NSCLC involves the intricate interplay between cell cycle regulatory proteins and tumor development. Among these proteins, Cyclin D1 (CCND1), Cyclin-dependent kinase 4 (CDK4), and p21 (also known as CDKN1A) play pivotal roles in controlling cell cycle progression and cellular proliferation.

CCND1, together with its partner CDK4, forms a critical complex that drives cells from G1 to S phase in the cell cycle, promoting cell proliferation (Qie and Diehl, 2016). The activity of CCND1-CDK4 complex is regulated by p21, which acts as a cyclin-dependent kinase inhibitor (Nardone et al., 2021). When p21 is upregulated, it binds to the CCND1-CDK4 complex and inhibits its kinase activity, effectively halting cell cycle progression. Consequently, p21 functions as a tumor suppressor by impeding uncontrolled cell proliferation.

Given the significance of the p21-mediated regulation of CCND1-CDK4 activity in suppressing tumor growth, it becomes imperative to explore novel therapeutic strategies aimed at targeting this specific pathway. Computer-aided drug design (CADD) has emerged as a powerful tool in the discovery of small molecule inhibitors (Luise and Robaa, 2018) that can modulate protein-protein interactions, such as the p21-CCND1-CDK4 axis. By computationally screening a large chemical space (Liu et al., 2021), CADD allows for the identification of potential drug candidates that could selectively bind to the target proteins and disrupt their functional interactions (Danel et al., 2023).

The successful application of CADD in drug discovery has revolutionized cancer treatment (Sobral et al., 2023), leading to the development of several approved therapies targeting various cancers. For instance, in breast cancer, the FDA-approved CDK4/6 inhibitors, such as palbociclib (Lee et al., 2022), have demonstrated substantial clinical benefits by inhibiting the activity of CCND1-CDK4 complex and arresting tumor growth. Moreover, in melanoma, the combination therapy of p21-activating kinase (PAK) inhibitors and immunotherapies has shown remarkable efficacy by overcoming tumor resistance and enhancing the anti-tumor immune response (Ma et al., 2022).

In this study, we aim to elucidate the relationship between CCND1, CDK4, p21, and NSCLC, with a specific focus on the mechanistic role of p21 in inhibiting CCND1-CDK4 complex activity. Leveraging computer-aided drug design, we will explore the potential of designing novel therapeutic agents that could modulate the p21-CCND1-CDK4 axis, offering new and exciting avenues for targeted therapy in NSCLC. By harnessing the power of computational methods, we strive to contribute to the development of more effective and personalized treatments for NSCLC and other malignancies.

## 2 Method

### 2.1 Protein preparation

The 3D structure of CCND1-CDK4-P21 (6P8H) (Guiley et al., 2019) CCND1-CDK4 (2W9Z) (Day et al., 2009) were downloaded from the RCSB PDB database (https://www.rcsb.org/). Before docking, the Protein Preparation Wizard of Maestro was used for protein preparation. This process included the protonation of atoms, removal of water molecules, fixation of atoms, and an energy minimization step using OPLS4 at pH 7 ± 2. Specifically, the PROPKA program was employed to predict the pKa values of the protein residues. By selecting “Use PROPKA” and “Label pKas” in the Refine tab, residues were labeled with their respective pKa values. The optimization of hydrogen bond networks included altering residue ionization and tautomer states, considering relative penalties for different protonation states derived from pKa estimates. Additionally, the Interactive Optimizer was used to manually adjust the protonation state of individual residues if necessary, refining the protonation states further based on specific requirements or observations from the simulation.

### 2.2 Gromacs MD simulation

Part of molecular dynamics (MD) simulations presented in this study were conducted using the GROMACS 23.1 package (https://www.gromacs.org/). The AMBER 99SB-ILDN (Lindorff-Larsen et al., 2010) and explicit solvation were employed, and each system was placed in a rectangular box of SPC water molecules with a minimum distance of 10Å between any solute atom and the edges of the periodic box. Counter ions were added to neutralize the total charge of the system. The system underwent an energy minimization process using the steepest descent method, with the maximum set to 1000.0 kJmol^−1^nm^−1^. Subsequently, the system was equilibrated in two steps: (1) canonical ensemble (NVT, 1 ns) and (2) isothermal–isobaric ensemble (NPT, 1 ns). Following equilibration, the MD simulations were run for 500ns. To ensure numerical stability, all bonds involving hydrogen atoms were constrained using the default linear constraint solver algorithm (LINCS) (Hess, 2008). The Vrescale thermostat and Parrinello–Rahman barostat were utilized with the temperature set at 300K and pressure at 1.0bar, with time constants of 0.1 and 2ps, respectively. The Particle-Mesh Ewald (PME) method was employed to handle long-range interactions, and a 10Å cutoff was utilized for van der Waals interactions (Darden et al., 1993). The time step was set to 2 fs, and a snapshot was collected every 1.0ps.The topology files for the 513457 was obtained from Sobtop (Tian Lu, Sobtop, Version 1.0, http://sobereva.com/soft/Sobtop)

Based on the results of MD simulation, we calculated Root Mean Square Deviation (RMSD); Root Mean Square Fluctuation (RMSF), Radius of gyration (Rg), and hydrogen bond interactions, hydrogen bond autocorrelation function (C(t)) (Dixit and Taniguchi, 2023), Principal Component Analysis (PCA) (Campitelli et al., 2021; Chen et al., 2021; Bao et al., 2023), Free Energy Landscape (FEL) (Malmstrom et al., 2015), and vector movements. Dynamic cross-correlation matrices (DCCM) (Ghosh and Vishveshwara, 2007) were drawn byBio3D (Grant et al., 2006). RMSD was calculated using C-alpha atoms. For RMSF, PCA, and DCCM, residue positions were used. RMSD, RMSF, Gg, H-bond, and FEL results were visualized by Matplotlib (https://matplotlib.org/).

### 2.3 Atom-based 3D-QSAR modelling and screening

Receptor-ligand complex based pharmacophore model was developed using PHASE (Dixon et al., 2006) module of Schrödinger 2023-2 software. PHASE has a built-in set of six pharmacophore features, i.e., hydrogen-bond acceptor (A), hydrogen-bond donor (D), hydrophobic group (H), negatively ionizable (N), positively ionizable (P), and aromatic ring (R). 1,640,000 compounds were retrieved from TargetMol (https://www.targetmol.cn/). The compounds were added to a phase database and screened based on the selected pharmacophore models.

### 2.4 Desmond MD simulation

Part of molecular dynamics (MD) simulations presented in this study were conducted using the Desmond package in the Schrödinger 2023-2 software. Firstly, 200 ns MD simulation was run for the top 20 compounds with the best binding affinity to CCND1, then the best four stable complex proceeded for 500 ns simulation time. TIP3P water model was used for system builder, and orthorhombic box shape was assigned with distances 10 Å and force filed OPLS4. Then ions were neutralized by additions of required charges. During MD simulation at each 500ps interval, snapshots were recorded, the nose-hover thermostat method was specified with a relaxation time of 1ps and 2fs time step. The system was minimized at 2000 iterations, NPT temperature was at 300 K, then Maestro was used for visualization of trajectories.

### 2.5 Protein docking

HDOCK (http://hdock.phys.hust.edu.cn/), a hybrid algorithm of template-based modeling and *ab initio* free docking (Yan et al., 2017), was employed to execute molecular docking between the 513457-effected CCND1 and CDK4. The workflow of HDOCK consists of five steps: (1) uploading of input receptor and ligand molecules in the PDB file format, (2) selection of the best receptor (HHSearch) and ligand (FASTA) templates by sequence similarity search against the PDB sequence database, (3) structure homology modeling using MODELLER for receptor and ligand, (4) fast Fourier transform (FFT) based HDOCK lite global docking, and (5) visualization of docking models and template-based model as an output (Huang and Zou, 2008; Yan and Huang, 2020).

## 3 Results

### 3.1 Initializing the structure of CCND1 through molecular dynamics simulations

**FIGURE 1:**
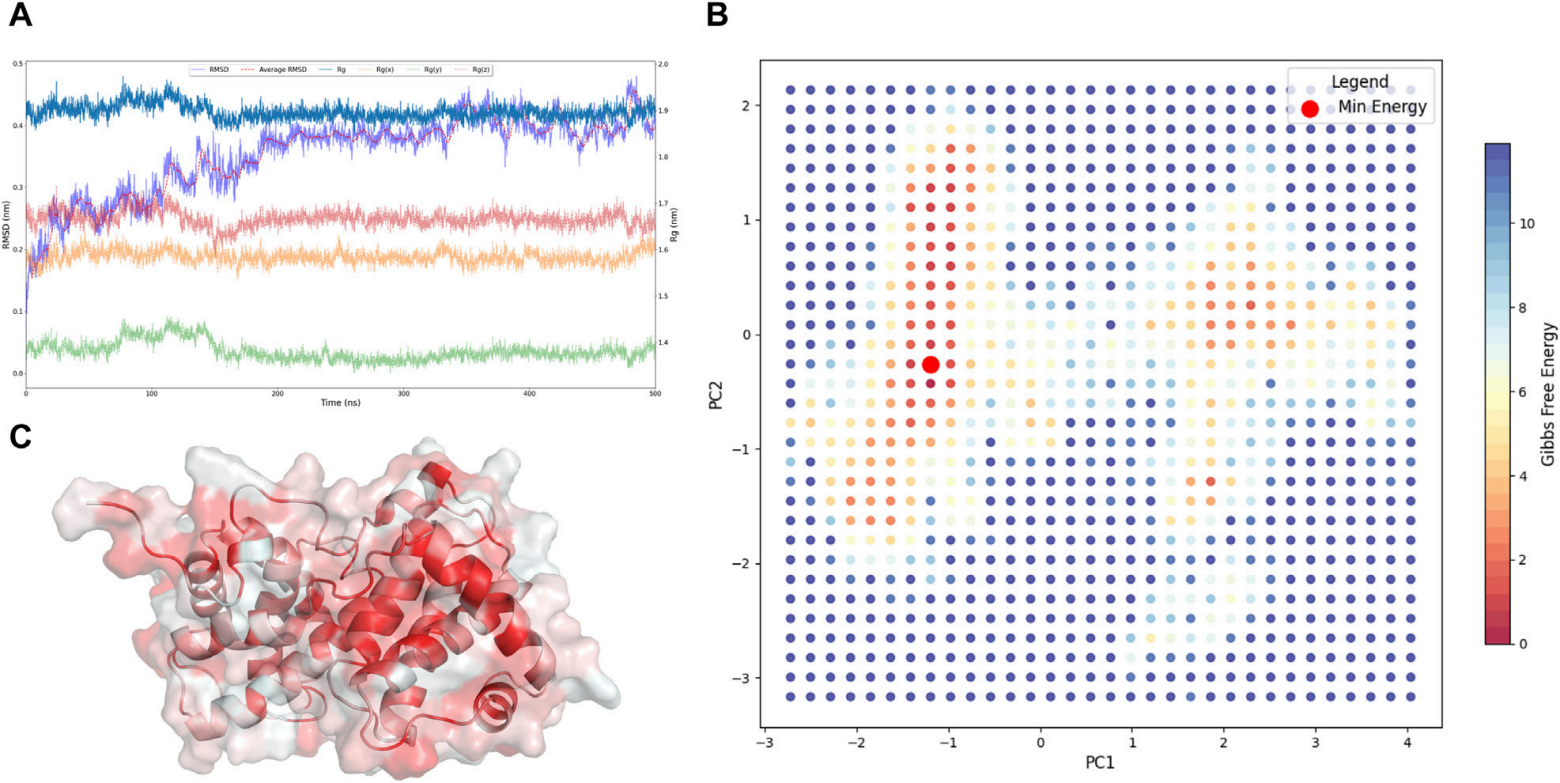
Acquisition of the Resting State of CCND1. **(A)** Dynamic changes of RMSD and Rg of CCND1 during the simulation process. **(B)** 2D free energy landscape constructed based on PC1, PC2, and Gibbs free energy. **(C)** Lowest energy conformation of CCND1 obtained from the free energy landscape.

In the presented investigation, we conducted a comprehensive molecular dynamics simulation of CCND1 in its apo form (An et al., 2019), derived from the crystal structure of CCND1 in complex with CDK4-P21 (Guiley et al., 2019). The analysis, which spanned over a 500 ns timeframe, was instrumental in observing system stability through the critical parameters: RMSD and Rg (Yu et al., 2023), including the distribution across the x, y, and *z*-axes (Figure 1A).

The RMSD trajectory initiated at 0 nm and progressively climbed to a peak of 0.41 nm at the conclusion of the simulation, indicating the structural variations occurring throughout the simulation. Notably, a plateau reached post-200 ns attested to a stabilized system. Concurrently, the Rg and its axial components Rg(x), Rg(y), and Rg(z) displayed comparable trajectories. The Rg, commencing at 1.89 nm, underwent mild oscillations, ultimately settling at 1.93 nm at the 500 ns mark, reflecting minor modifications in the system’s compactness.

To substantiate our claims of comprehensive sampling of CCND1 apo, we executed PCA (Fu et al., 2023) on the simulation trajectory and calculated the Gibbs free energy at various time points. Given that PC1 and PC2 encapsulated 48% of the dynamical changes during the CCND1 simulation, as shown in Supplementary Figure S1, these principal components were considered to be aptly representative of significant dynamical variations (Nyamai and Bishop, 2020). Further calculations of the Gibbs free energy, based on PC1 and PC2, facilitated the construction of a free energy landscape for CCND1 apo (Figure 1B). This landscape revealed that the conformations with the lowest energy first appeared at 189 ns and last at 474.3 ns, substantiating our claim of comprehensive sampling of CCND1 based on the RMSD and Rg analyses.

Importantly, the identification of these low-energy conformations (Figure 1C) had immense practical implications. They served as the most stable structures of CCND1 in its apo state, making them particularly suitable for subsequent virtual screening studies. Utilizing these conformations as target structures could enhance the accuracy and efficiency of the virtual screening process, potentially accelerating the discovery of novel ligands or drugs (Zheng et al., 2017).

In summary, the concurrent trends in RMSD and Rg values over the 500 ns simulation period attested to satisfactory conformational sampling of the CCND1 apo system. These findings confirmed system stabilization and served as a solid foundation for additional structural and functional analyses, including potential applications in virtual screening strategies (Errasti-Murugarren et al., 2021).

### 3.2 Analysis of the dynamic mechanism of P21 inhibition on the CCND1-CDK4 complex

To shed light on the inhibitory mechanism that P21 exerts on the CCND1-CDK4 complex, a 500 ns molecular dynamics simulation for both CCND1-CDK4 (Day et al., 2009) and CCND1-CDK4-P21 was performed. The dynamic shifts in RMSD and Rg during the simulation, suggesting a substantial conformational stability of the complexes. The PCA and eigenvector proportions for both simulations are illustrated in Supplementary Figures S2, S3. With the sum of PC1 and PC2 exceeding 50% in both simulations, the data indicates a high level of representativeness and hence suitability for further evaluation.

**FIGURE 2:**
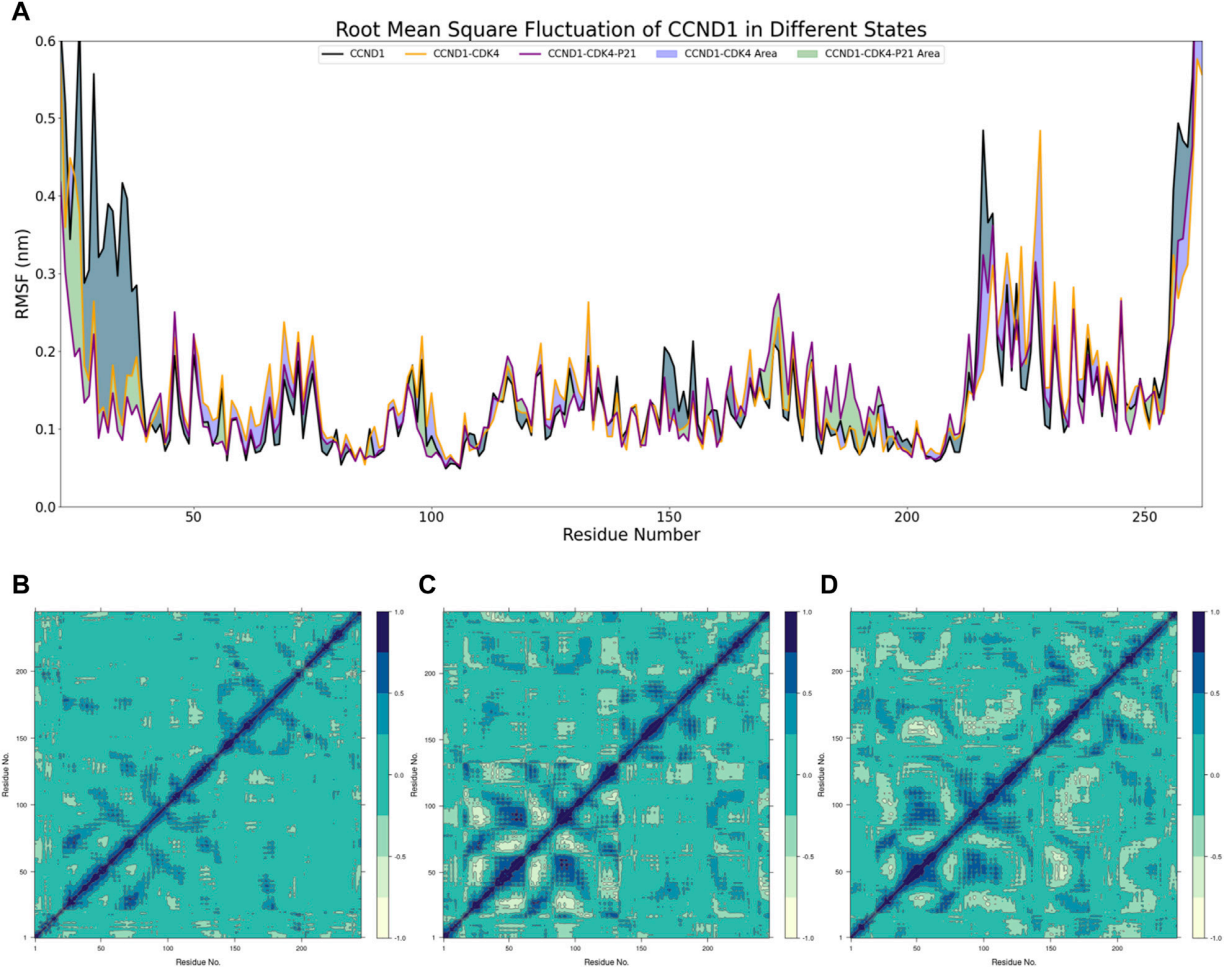
Dynamic Mechanism Analysis of P21 Inhibition on the CCND1-CDK4 Complex. **(A)** RMSF Analysis: Different colors represent the distribution of RMSF across residues for different structures, and the colored blocks illustrate the differences in RMSF among different structures. **(B)** Covariance Matrix of CCND1 over 500 ns: Positive values indicate positive correlated motions between residues, while negative values indicate anticorrelated motions between residues. **(C)** Covariance Matrix of CCND1-CDK4 over 500 ns: Positive values indicate positive correlated motions between residues, while negative values indicate anticorrelated motions between residues. **(D)** Covariance Matrix of CCND1-CDK4-P21 over 500 ns: Positive values indicate positive correlated motions between residues, while negative values indicate anticorrelated motions between residues.

With this foundation, the RMSF of CCND1 was assessed under three distinct states, as delineated in Figure 2A. This analysis highlighted that the N- and C- termini of CCND1 were subjected to diverse degrees of restriction upon their interaction with CDK4, thereby enhancing the structural stability of the CCND1-CDK4 complex. The 50-150 and 220-240 peptide segments demonstrated a range of RMSF increases, indicating these areas of CCND1 structural flexibility are pivotal for the successful binding and dynamic stability with CDK4. Upon the engagement with P21, RMSF was curtailed to varying extents within the 50-130 peptide segment. As a result, we inferred that P21 chiefly curtails the activity of the CCND1-CDK4 complex by restraining the flexibility of this particular peptide segment.

Moreover, DCCMs of CCND1 under varying conditions were composed to scrutinize the dynamic characteristics within the CCND1 molecule (Biagini et al., 2022), as depicted in Figures 2B–D. Due to a technical limitation in Bio3D, all residues were indexed from 1, while in reality, each DCCM started from residue 22. Observations revealed that the positively and negatively correlated movements within the 40-150 peptide segment were constrained to differing extents after the engagement of P21 with the CCND1-CDK4 complex. Conversely, the negatively correlated movements between the 50-130 and 170-260 peptide segments intensified, aligning with the peptide segment regions influenced by P21 as disclosed in the RMSF analysis. Hence, the peptide segment spanning from 50 to 130 may serve as the primary region where P21 curtails the activity of the CDK4-CCND1 complex.

**FIGURE 3:**
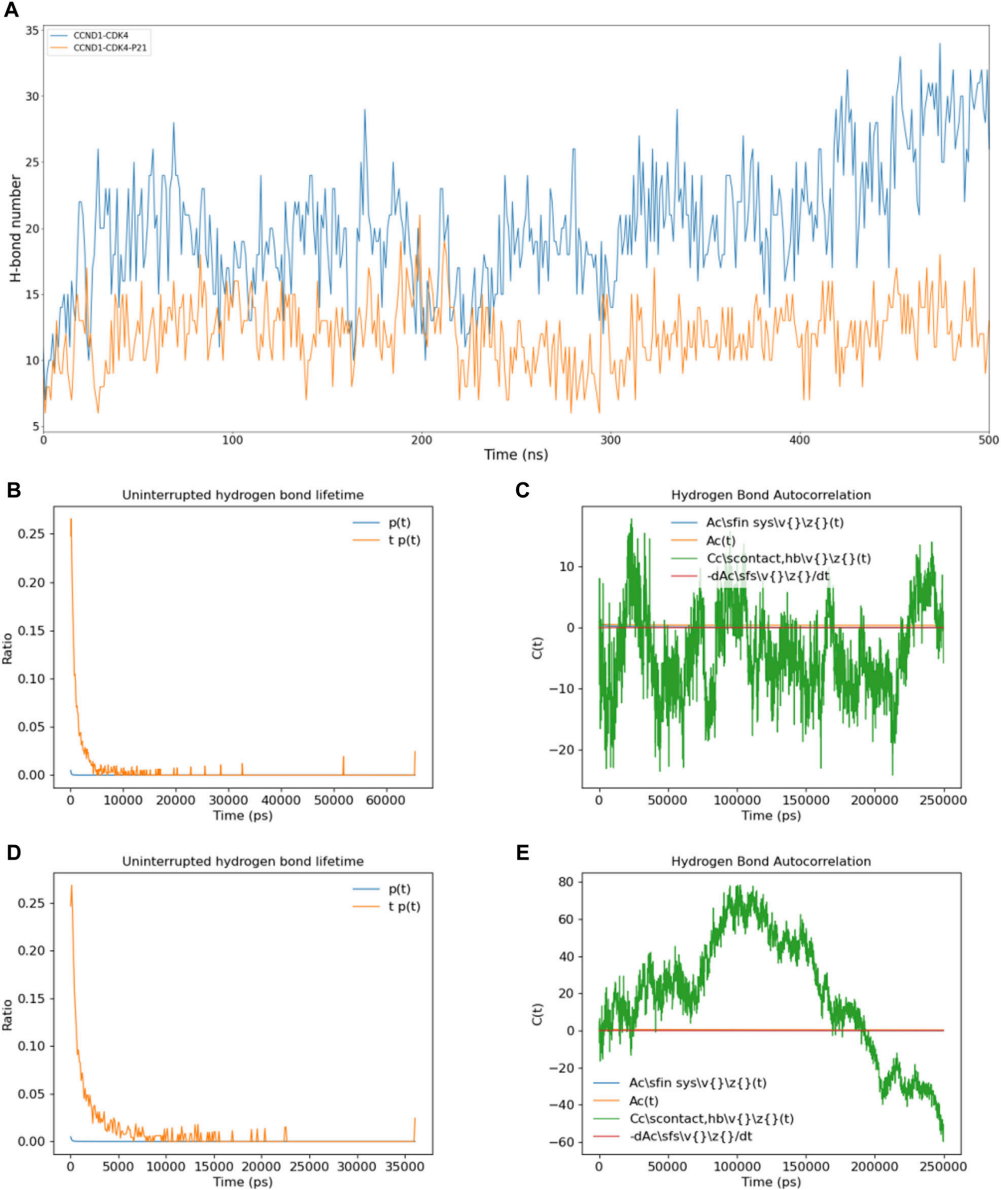
Impact of P21 on Intermolecular Hydrogen Bonds in the CCND1-CDK4 Complex. **(A)** Changes in the number of intermolecular hydrogen bonds between CCND1 and CDK4 in the CCND1-CDK4 and CCND1-CDK4-P21 complexes over time. **(B, C)** Distribution of the lifetimes of intermolecular hydrogen bonds between CCND1 and CDK4 in the CCND1-CDK4 and CCND1-CDK4-P21 complexes. **(D, E)** Autocorrelation functions of intermolecular hydrogen bonds between CCND1 and CDK4 in the CCND1-CDK4 and CCND1-CDK4-P21 complexes. Note that the 250 ns corresponds to the normal value calculated during the 500 ns simulation process.

In a bid to unravel the dynamic shifts in intermolecular interactions between CCND1 and CDK4 in the presence and absence of P21’s involvement with the CCND1-CDK4 complex, we conducted a series of calculations. These included quantifying the count of intermolecular hydrogen bonds between CCND1 and CDK4 (Figure 3A), ascertaining the persistence duration of these intermolecular hydrogen bonds (Figures 3B, C), and determining the autocorrelation function of the intermolecular hydrogen bonds (Figures 3D,E).

Comparisons were drawn regarding the count of intermolecular hydrogen bonds between CCND1 and CDK4, pre and post the attachment of P21. This comparison clearly indicated P21’s role in effectively restraining the quantity of intermolecular hydrogen bonds, thus impairing the binding stability between the two entities. Subsequent duration and autocorrelation function analysis of the intermolecular hydrogen bonds lent support to P21’s disruptive influence over the intermolecular hydrogen bonds between CCND1 and CDK4 from a temporal perspective (Naz et al., 2019).

During the phase preceding P21’s attachment, the hydrogen bonds between CCND1 and CDK4 could be sustained for as long as 65 ns. Conversely, following P21’s attachment, this duration witnessed a significant curtailment, with the longest persistence only lasting for 37 ns. The autocorrelation function analysis of intermolecular hydrogen bonds exhibited constant fluctuations in the phase preceding P21’s involvement. This signified a continual cycle of formation of new hydrogen bonds and rupture of old ones during the simulation, thus illustrating the dynamic nature of the thermal motion within the complex (Lentz and Garofalini, 2018).

However, following the involvement of P21, the autocorrelation function of hydrogen bonds between CCND1 and CDK4 embarked on a consistent descent beginning at 100 ns, denoting a steady reduction in intermolecular hydrogen bonds. In summary, the dynamics of P21’s activity constraining the CCND1-CDK4 complex manifested in the form of reduced stability of the intermolecular hydrogen bonds and a diminished count of hydrogen bonds formed between the two entities.

### 3.3 Pharmacophore generation and virtual screening based on partial peptide segments of P21

To discover compounds with P21-like inhibitory effects on CCND1, we utilized a pharmacophore model based on the P21-CCND1 loop structure (Supplementary Figure S4) for virtual screening of 1.63 million compounds. Guided by MMGBSA scoring (Tuccinardi, 2021), we selected compounds with scores below −60 kcal/mol (Table 1).

**TABLE 1:** Scoring and interacting residues of the selected compounds.

Compound ID	MMGBSA	Interacted residues			
108586	−61.04	Gln-100	Tyr-127		
203037	−60.07	Gln-100	Tyr-127		
221050	−60.79	Trp-63	Glu-66	Glu-70	Tyr-127
329311	−60.69	Trp-63	Gln-100	Tyr-127	
513457	−60.87	Gln-100	Tyr-127		
724885	−60.67	Trp-63	Gln-100	Tyr-127	
852175	−64.05	Gln-100			
914014	−60.99	Trp-63	Gln-100		
1073277	−63.93	Trp-63	Tyr-127		
1081460	−63.3	Trp-63	Glu-66	Glu-70	Gln-100
1124644	−62.31	Trp-63	Gln-100	Tyr-127	
1127615	−61.66	Trp-63	Gln-100	Tyr-127	
1218445	−60.31	Trp-63	Gln-100	Tyr-127	
1255883	−62.56	Trp-63	Glu-66	Tyr-127	
1287941	−62.22	Trp-63	Gln-100	Tyr-127	
1302891	−63.03	Trp-63	Gln-100	Tyr-127	
1326859	−63.74	Trp-63	Gln-100	Ile-126	
1336634	−60.35	Trp-63	Gln-100	Tyr-127	
1347373	−60.43	Trp-63	Gln-100		
1478579	−61.85	Gln-100			

Notably, all compounds exhibited MMGBSA values below −60 kcal/mol, indicating strong binding affinities with CCND1. Compound 852175 stood out with the highest MMGBSA value (−64.05 kcal/mol), representing an exceptionally favorable binding affinity. Conversely, compound 203037 displayed the lowest MMGBSA value (−60.07 kcal/mol), indicating a comparatively less potent but still favorable binding affinity.

Interactions with common residues, such as Gln-100 and Tyr-127, were observed in compounds 108586, 203037, and 513457. Additionally, Trp-63 and Gln-100 were recurrently involved in compounds 329311, 724885, and 914014. However, the interactions varied among compounds. Some compounds interacted solely with Gln-100 (852175 and 1478579), while others engaged with multiple residues, such as compounds 221050 and 1081460, which interacted with four residues each (Trp-63, Glu-66, Glu-70, and either Tyr-127 or Gln-100, respectively). Compound 1326859 demonstrated unique interactions with Trp-63, Gln-100, and Ile-126, distinguishing it from other compounds in the table.

These findings underscore the diverse interactions and potential inhibitory effects of the selected compounds on CCND1-CDK4. Our virtual screening approach provides valuable insights and holds promise for identifying potential therapeutic agents. Similar techniques have proven successful in different cancers drug discovery (Okamoto et al., 2011; Zolek et al., 2023), leading to the development of effective anti-cancer drugs. Likewise, our approach may offer new avenues for cancer treatment by targeting CCND1-CDK4.

### 3.4 Binding stability analysis based on molecular dynamics simulation

**FIGURE 4:**
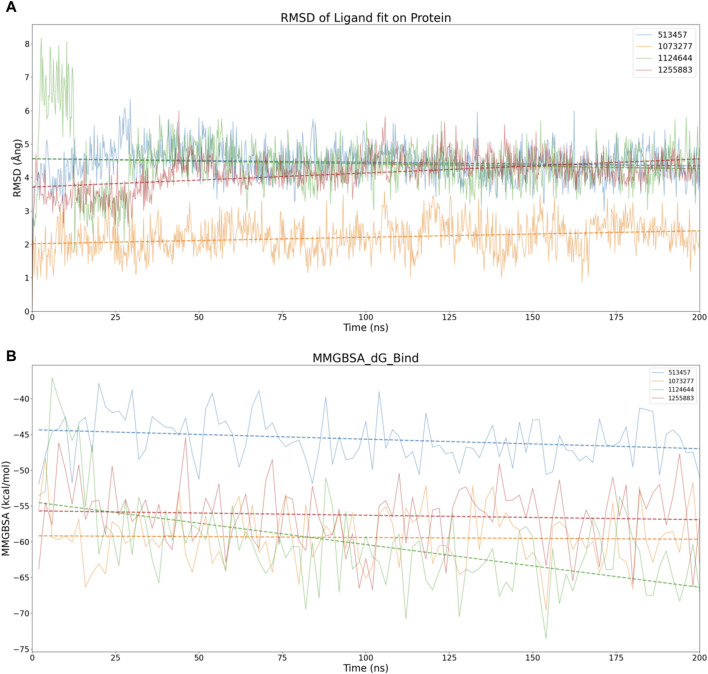
Stability analysis of the structural and energetic aspects at 200 ns timescale. **(A)** Dynamic changes of RMSD over time, with dashed lines representing trend lines for the dynamic changes of RMSD for different compounds. **(B)** Dynamic changes of MMGBSA over time, with dashed lines representing trend lines for the dynamic changes of MMGBSA for different compounds.

Considering the virtual screening strategy we adopted involved semi-flexible docking, where the docking region was designated as a rigid area, assessing the stability of the bond between the target and the compounds was not feasible. Therefore, we first performed 200 ns molecular dynamic simulations on 20 compounds, preliminarily evaluating the stability of the complexes via Ligand fit on Protein RMSD, depicted in Supplementary Figure S5. If the RMSD fluctuation range remained within 1-2 Ång, the simulation system was deemed to have achieved initial stability (Koska et al., 2008). According to this standard, we inferred that compounds 513457, 1073277, 1124644, and 1255883 may exhibit stable binding with CCND1(Rathod et al., 2023), as illustrated in Figure 4A.

To conduct a dimensional reduction analysis of RMSD, we charted trend lines of RMSD’s dynamic changes over time for these four compounds. Interestingly, both 1073277 and 1255883 exhibited an upward trend, while 513457 and 1124644 maintained a flat line, suggesting the possible occurrence of off-target effects with 1073277 and 1255883 (Giatti et al., 2021).

Considering other angles to analyze the potential for off-target effects with 1073277 and 1255883, we calculated the MMGBSA dynamic changes over the 200 ns simulation period for the four compounds, as shown in Figure 4B. Contrary to expectations, none of the four compounds showed an upward trend in MMGBSA values. Instead, they seemed to continually enhance their affinity with CCND1 through thermodynamic motion. Thus, we initially speculate that the upward trend in RMSD exhibited by 1073277 and 1255883 might represent these compounds undergoing conformational adjustments, transitioning towards optimal binding conformations. However, due to the relatively short simulation time, we still need to extend the simulation time for these four sets of compounds.

**FIGURE 5:**
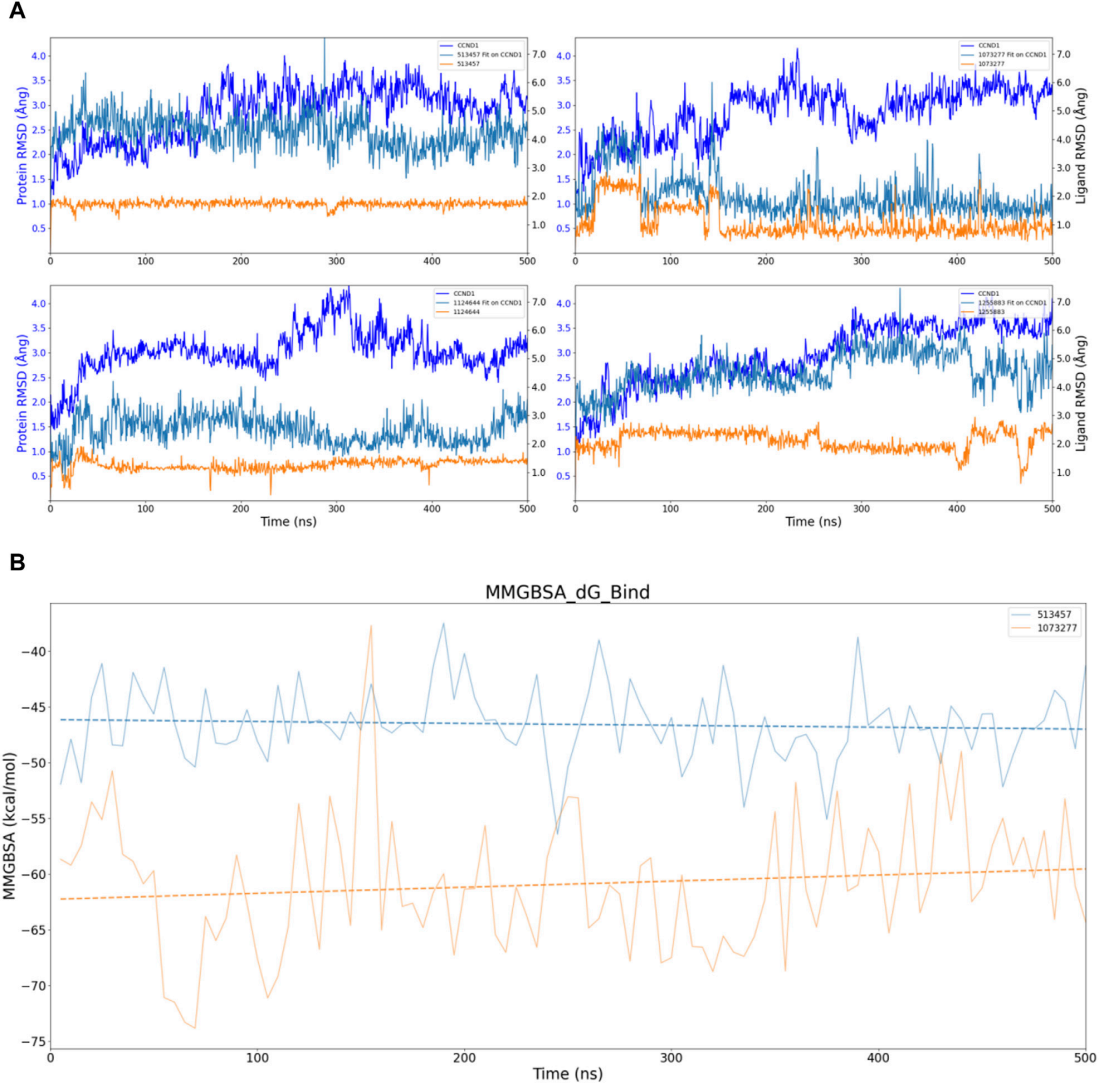
Stability analysis of the structural and energetic aspects at 500 ns timescale. **(A)** Dynamic changes of RMSD over time, with dashed lines representing trend lines for the dynamic changes of RMSD for different compounds. **(B)** Dynamic changes of MMGBSA over time, with dashed lines representing trend lines for the dynamic changes of MMGBSA for different compounds.

In pursuit of a more comprehensive analysis, we extended the simulations to 500 ns for four sets of compounds, adding an analysis of the protein’s own RMSD and the compound’s own RMSD. As depicted in Figure 5A, considering the fluctuation threshold range of two Ång, only 513457 and 1073277 seemed to exhibit the kinetic characteristics of stable receptor-ligand binding after a series of conformational changes in the later stages of the simulation. Compared to 1073277, 513457 exhibited more pronounced fluctuations in its Fit on CCND1 RMSD, indicating weaker binding stability. However, when considering the compound’s own RMSD, the structural stability of 513457 far surpassed that of 1073277.

Taking into account the inherent properties of small molecular compounds, the fluctuation range of 1-2 Ång for 1073277 typically indicates certain conformational changes in the molecule, even surpassing three Ång at one point. This implies that it continually undergoes conformational changes to achieve stable binding with the pocket of CCND1. However, when 1073277 can no longer accommodate changes in the CCND1 pocket through its own conformational changes, there is a risk of off-target effects.

To address this, we also performed dynamic MMGBSA calculations for the two 500 ns simulations, as illustrated in Figure 5B. Through the trend lines, it is not difficult to discern that during the dynamic simulation process of the 513457-CCND1 complex, the binding energy between the two continually increases (inversely proportional to the numerical changes). In contrast, during the dynamic simulation of the 1073277-CCND1 complex, the binding energy exhibited a downward trend, further corroborating our previous speculation about the potential off-target risks associated with 1073277.

Upon executing 500 ns simulations for the four sets of compounds, only 513457 unveiled potential for enduring binding with CCND1. Consequently, the ensuing analysis will concentrate solely on the 513457-CCND1 complex.

### 3.5 Comparative protein-ligands contact analysis of 513457-CCND1 complex

**FIGURE 6:**
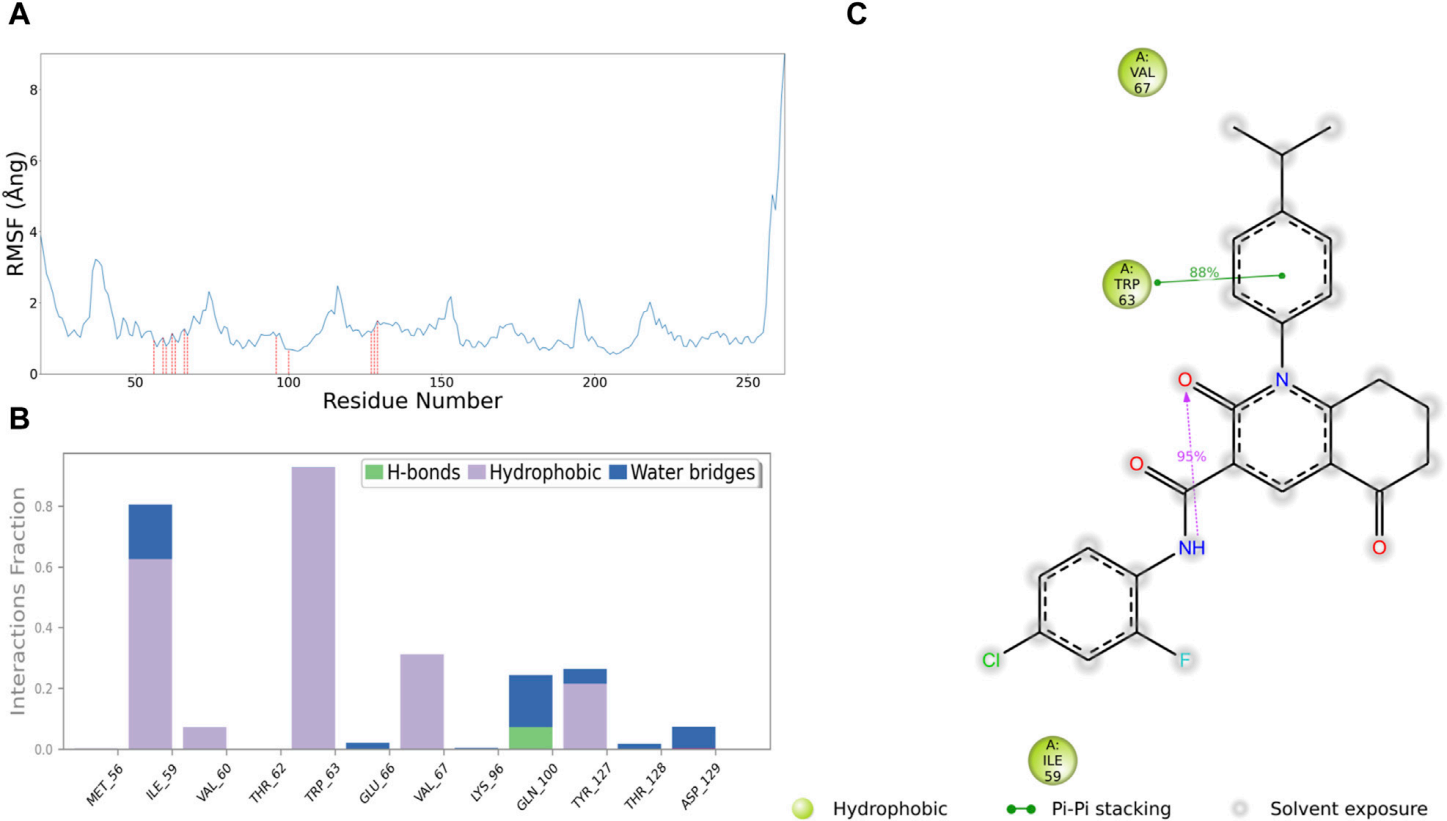
Interaction analysis between 513457 and CCND1. **(A)** Distribution of RMSF for different residues, with a red dashed line indicating residues that interact with 513457. **(B)** Types of interactions between different residues and 513457, along with the percentage of each interaction type during the simulation. **(C)** 2D plot illustrating specific interactions between CCND1 and 513457, displaying only interactions with a frequency exceeding 30%.

For further development of 513457, we conducted a series of analyses on the receptor-ligand interaction between 513457 and CCND1 in the simulation process. Figure 6A shows an array of amino acids, their engagement with the ligand, and the associated RMSF values (Yu et al., 2019). The amino acids ranged from Asp-19 to Ala-262. However, only a select few amino acids established contact with the ligand, namely, Met-56, Ile-59, Val-60, Thr-62, Trp-63, Glu-66, Val-67, Lys-96, Gln-100, Tyr-127, Thr-128, and Asp-129. These contacts had relatively low RMSF values, indicating a more stable interaction. On the other hand, the remaining amino acids did not engage with the ligand, and their RMSF values fluctuated between a minimum of 0.550 Ångand a maximum of 9.018 Ång. The pronounced RMSF values, especially towards the end of the sequence, indicated higher fluctuation and lower stability in those regions.

**FIGURE 7:**
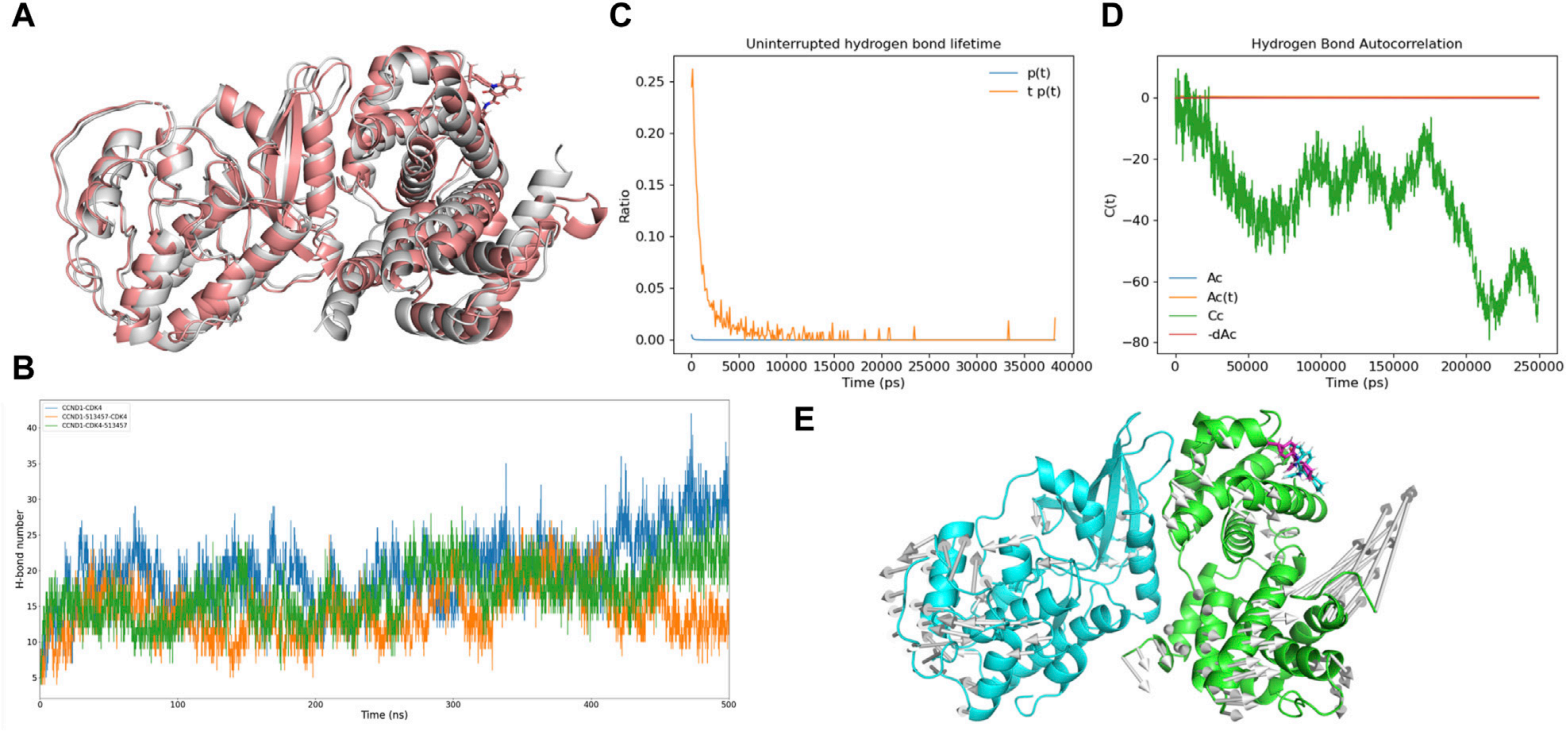
Analysis of the potential inhibitory mode of 513457. **(A)** Structural comparison of the complex formed by CCND1 with 513457 before and after binding to CDK4. The brick-red cartoon structure represents the complex formed by 513457 binding to CCND1 and CDK4, while the gray cartoon structure represents the crystal structure of the CCND1-CDK4 complex. **(B)** Dynamic changes in the number of hydrogen bonds formed between the complex formed by CCND1 with 513457 before and after binding to CDK4 and the crystal structure of the CCND1-CDK4 complex, as a function of time. **(C)** Distribution of hydrogen bond lifetimes between CCND1 and CDK4 after preferential binding of 513457 by CCNA1. **(D)** Autocorrelation function of hydrogen bonds between CCND1 and CDK4 after preferential binding of 513457 by CCND1. **(E)** The trend of residue motion during the simulation process.

In the subsequent interaction analysis, as shown in Figures 7B, C, we found that the interaction between 513457 and CCND1’s Met-56 and Trp-63 persisted throughout the simulation, indicating a highly stable structure-activity relationship between 513457 and these two residues (Zhao et al., 2016). Following that, Val-67, Gln-100, and Tyr-127 were identified, although their proportions were all below 0.4. However, by examining the Protein-Ligand Contacts count, as shown in the Supplementary Figure S7, it was evident that 513457 established contacts with Val-67, Gln-100, and Tyr-127 throughout the simulation, albeit at a slightly lower frequency than Met-56 and Trp-63.

### 3.6 Uncovering the potential mechanism of 513457 action

To investigate the potential of 513457 as a novel CCND1-CDK4 complex protein-protein interaction (PPI) inhibitor, we conducted a series of molecular dynamic simulations and docking experiments. Comparing the conformation of CCND1-513457 with CDK4 to the crystal structure of the CCND1-CDK4 complex (Porter et al., 2019), we observed significant conformational differences in the presence of 513457 (Figure 7A). Notably, 513457 reduced the number of hydrogen bonds between CCND1 and CDK4, a pattern consistent with the effects observed with P21 (Supplementary Figure S6). However, it is important to note that the docking results only permit comparisons before and after 513457’s binding to CCND1.

To explore an alternative mode of action, wherein 513457 binds to the pre-formed CCND1-CDK4 complex, we performed two sets of molecular dynamic simulations. The CCND1-513457-CDK4 model involved CCND1 binding to 513457 first and then to CDK4, while the CCND1-CDK4-513457 model involved CCND1 binding to CDK4 first and then to 513457. Figure 7B revealed that the hydrogen bond changes in CCND1-CDK4 and CCND1-CDK4-513457 were quite similar, but notable differences were observed with CCND1-513457-CDK4. Specifically, during the later stages of the simulation (around 450-500 ns), the three complexes displayed distinct variations in the number of hydrogen bonds, with CCND1-CDK4 forming more hydrogen bonds than CCND1-CDK4-513457 and CCND1-513457-CDK4. This finding indicates that 513457 can influence the interaction strength of both complexes, regardless of its binding order to CCND1 and CDK4, a dynamic characteristic akin to that influenced by P21. Intriguingly, binding to CCND1 before CDK4 resulted in a stronger inhibitory effect.

Furthermore, we conducted hydrogen bond half-life and autocorrelation coefficient analyses, specifically focusing on the scenario where 513457 preferentially binds to CCND1 (Figures 7C,D). The hydrogen bond half-life was notably lower than that of the CCND-CDK4 complex, and the autocorrelation coefficient trend resembled that of the complete inhibition of P21 binding to the CCND1-CDK4 complex. These findings suggest that 513457 effectively exerts a function similar to P21 in inhibiting the activity of the CCND1-CDK4 complex, thereby curbing tumor cell proliferation. Additionally, modevectors (Kagami et al., 2020) derived from the simulation trajectory revealed a dynamic dissociation of the dimer, with CCND1 and CDK4 moving in opposite directions upon 513457 binding (Figure 7E).

In conclusion, our theoretical calculations strongly indicate that 513457 holds great promise as a potential CCND1-CDK4 PPI inhibitor, capable of effectively replacing P21 and significantly limiting tumor cell proliferation, particularly in scenarios with lower P21 levels. These findings open up new avenues for potential therapeutic interventions targeting the CCND1-CDK4 complex in cancer treatment.

## 4 Discussion

Designing drugs targeting the CCND1-CDK4 complex presents several challenges and complexities. The formation of this complex involves intricate protein-protein interactions between CCND1, CDK4, and P21, forming a complex signaling network (Dey et al., 2023). Understanding and navigating these interactions are crucial to avoid potential side effects and adverse reactions (Zhong et al., 2019). In addition, ensuring target specificity is essential, as CDK4 plays a significant role in cell cycle regulation and has multiple essential functions within cells. However, the known CCND1 inhibitor, CMLD010509, does not achieve this specificity (Manier et al., 2017). The drugs must be carefully designed to specifically target the CCND1-CDK4 complex, avoiding interference with other critical cell cycle proteins and minimizing potential toxic side effects (Nasrollahzadeh et al., 2020).

In this study, we aimed to investigate the inhibitory mechanism of P21 on the CCND1-CDK4 complex using MD simulations. Additionally, we performed virtual screening to identify potential compounds with P21-like inhibitory effects on CCND1 and further analyzed the binding stability of selected compounds with CCND1 through MD simulations.

The MD simulation results for CCND1 in its apo form provided a stable foundation for our subsequent analyses (Brewitz et al., 2023). By delving into the dynamic mechanism of P21 inhibition on the CCND1-CDK4 complex, we uncovered intriguing insights. P21 primarily restricts the flexibility of the 50-130 peptide segment, leading to a decrease in the activity of the CCND1-CDK4 complex. Additionally, P21 disrupts intermolecular hydrogen bonds between CCND1 and CDK4, further compromising the binding stability between the two proteins. These findings deepen our understanding of the inhibitory mechanism of P21 and shed light on potential avenues for designing targeted therapies (Bartling et al., 2021).

To identify potential compounds with P21-like effects on CCND1, we employed a pharmacophore model based on the P21 loop structure. This approach enabled us to identify several compounds exhibiting strong binding affinities to CCND1, thereby suggesting their potential as promising candidates for developing anti-tumor drugs. The selection process was meticulously validated through extensive MD simulations to assess the binding stability between these compounds and CCND1.

Among the compounds tested, 513457 emerged as a standout performer, displaying enduring binding with CCND1 over an extended period. This observation makes 513457 an attractive and robust candidate worthy of further exploration and development. Notably, further analysis of the 513457-CCND1 complex uncovered stable interactions with specific amino acids, such as Met-56 and Trp-63. These findings underscore a strong structure-activity relationship (Kirchmair et al., 2012), enhancing our confidence in 513457’s potential as an effective inhibitor of the CCND1-CDK4 complex protein-protein interaction.

Moreover, through in-depth molecular dynamic simulations and docking studies, we gained valuable insights into the potential inhibitory mode of 513457. Our findings indicate that this compound can effectively disrupt the interactions between CCND1 and CDK4, leading to inhibition of tumor cell proliferation. These exciting results highlight the promise of 513457 as a potential anti-tumor drug, particularly in scenarios where P21 levels are lower, pointing towards its potential to address critical unmet medical needs in cancer treatment.

The innovative design of our research approach, integrating MD simulations, virtual screening, and detailed binding stability analyses, provides a valuable framework for identifying and characterizing potential anti-tumor drugs targeting CCND1-CDK4 interactions. By elucidating the inhibitory mechanism of P21 and uncovering the exceptional properties of 513457, our study contributes to the advancement of cancer research and drug development. Future studies can leverage these findings to optimize 513457 and explore its therapeutic efficacy in preclinical and clinical settings, ultimately bringing us closer to more effective and targeted treatments for cancer patients. The combination of molecular dynamics simulations and virtual screening holds great promise for accelerating drug discovery and design, and our work provides valuable insights into the development of innovative and potent anti-cancer therapeutics.

## 5 Conclusion

In this study, we employed molecular dynamics simulations and computational drug design to investigate the crucial interactions between CCND1, CDK4, and p21 in the context of non-small cell lung cancer (NSCLC). Our findings reveal that p21 exerts its inhibitory effect on the CCND1-CDK4 complex by curtailing the flexibility of specific peptide segments. Moreover, we identified a potential novel inhibitor, compound 513457, which disrupts CCND1-CDK4 interactions similarly to p21, offering a promising avenue for targeted therapy in NSCLC. These results illuminate the intricate dynamics of this critical pathway and provide new insights into potential therapeutic interventions for NSCLC.

## Data Availability

The raw data supporting the conclusion of this article will be made available by the authors, without undue reservation.
