# Supplementary figures and images for: In silico discovery of potential PPI inhibitors for anti-lung cancer activity by targeting the CCND1-CDK4 complex via the P21 inhibition mechanism
Source: Front Chem. 2024 Jun 18;12:1404573. doi: 10.3389/fchem.2024.1404573 (PMC11217521; doi:10.3389/fchem.2024.1404573)

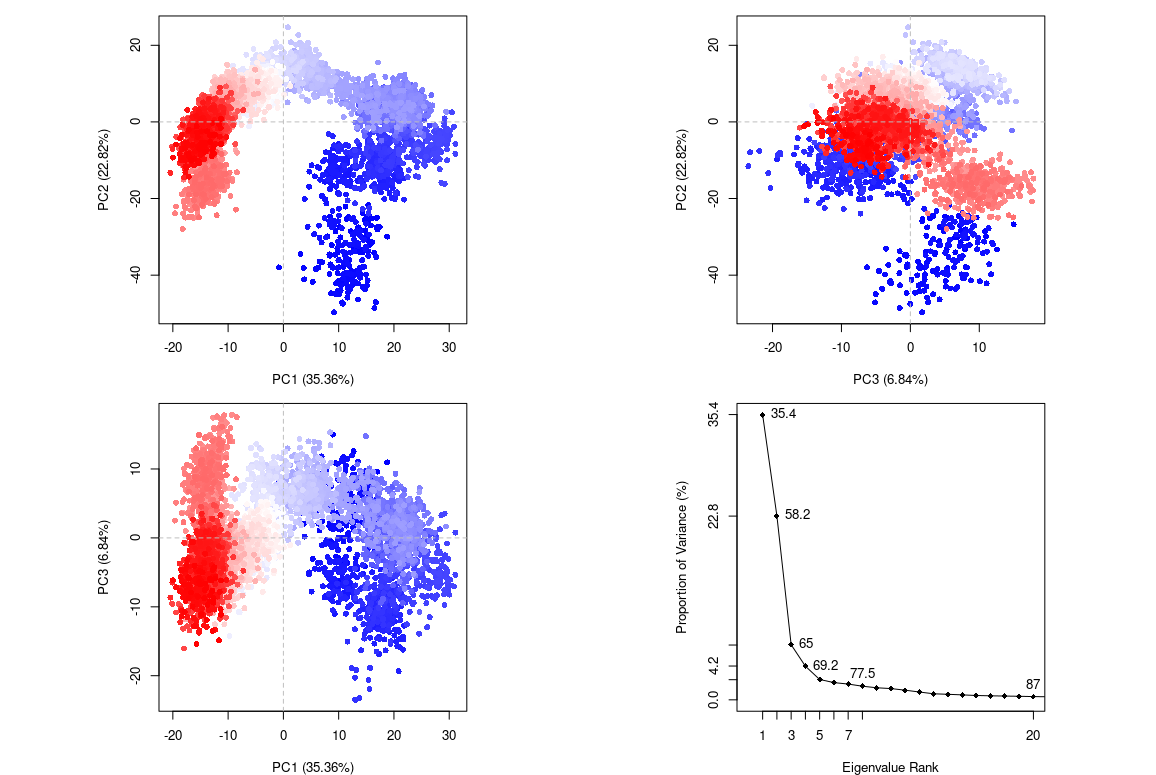

Supplement: Supplementary file 1 [file Image3.TIFF]

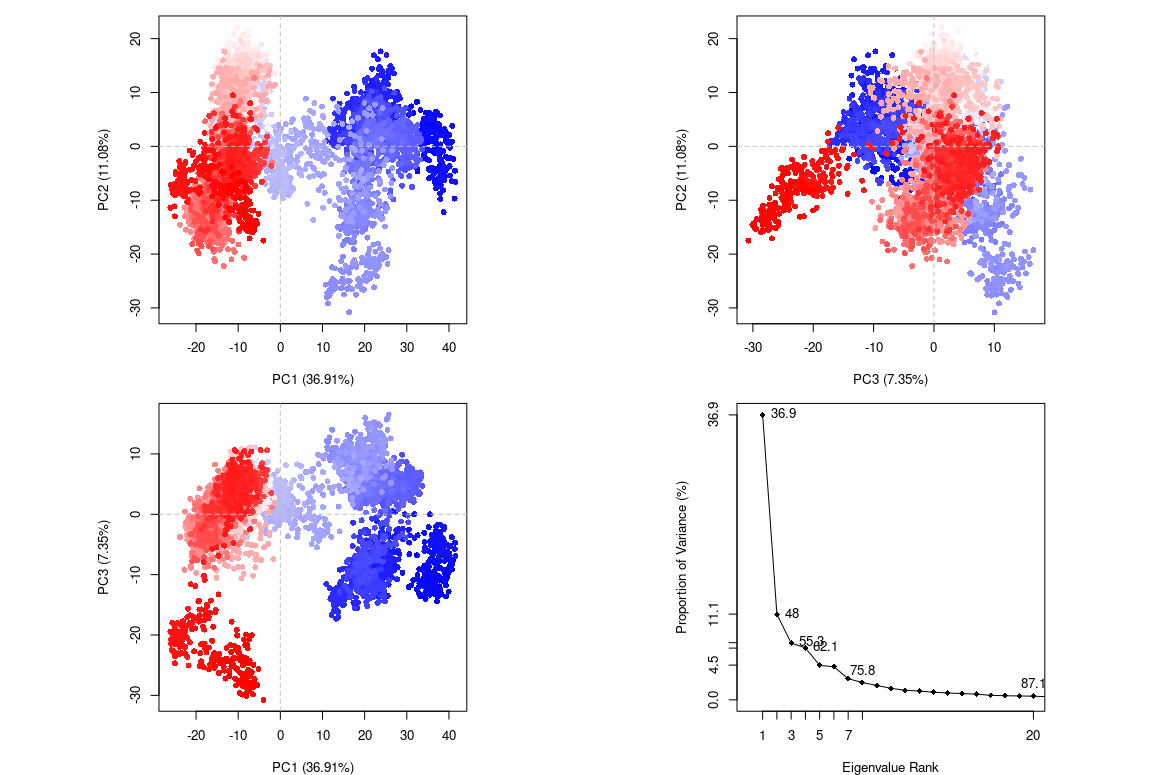

Supplement: Supplementary file 2 [file Image1.TIFF]

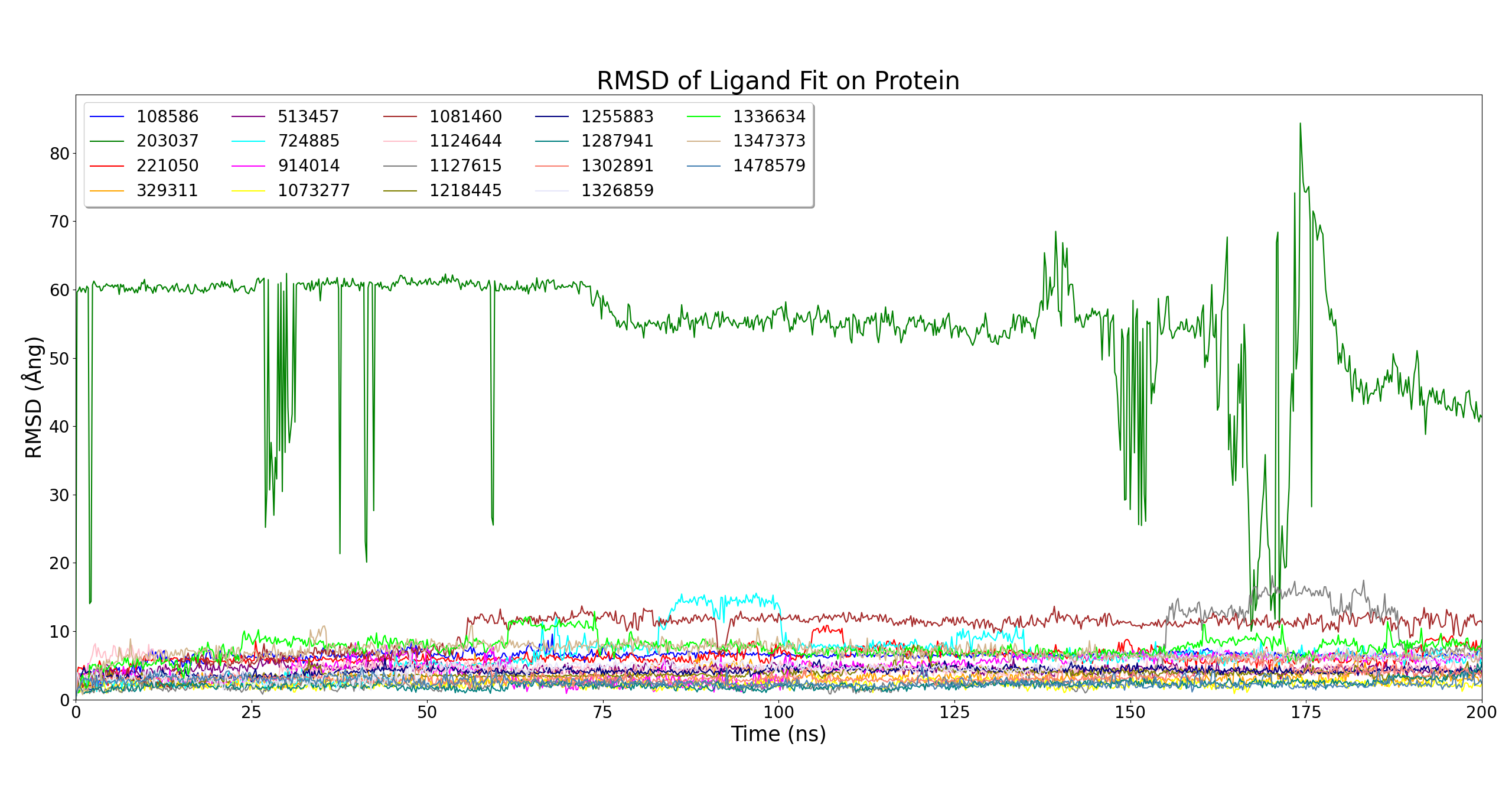

Supplement: Supplementary file 3 [file Image5.PNG]

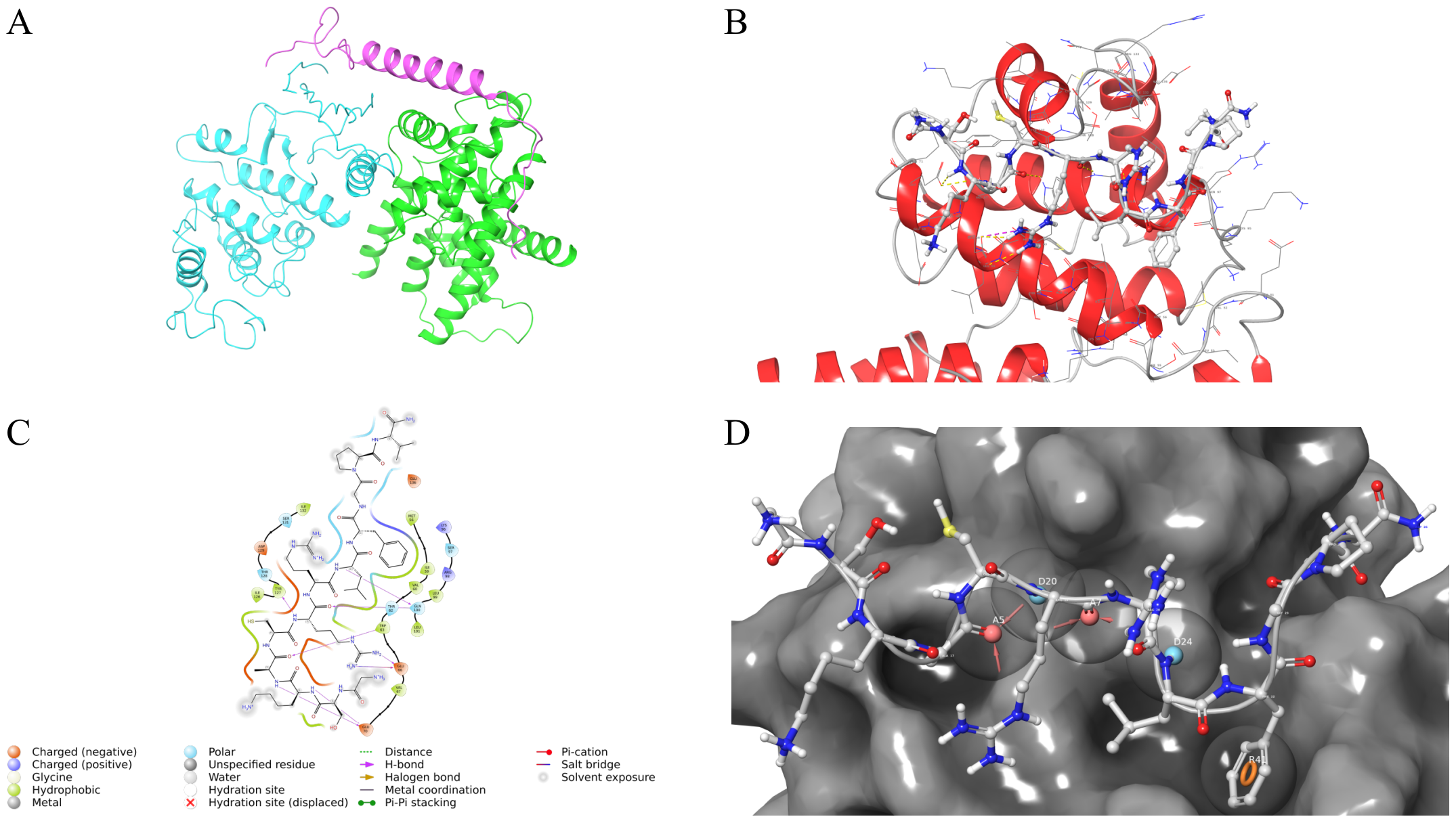

Supplement: Supplementary file 4 [file Image4.PNG]

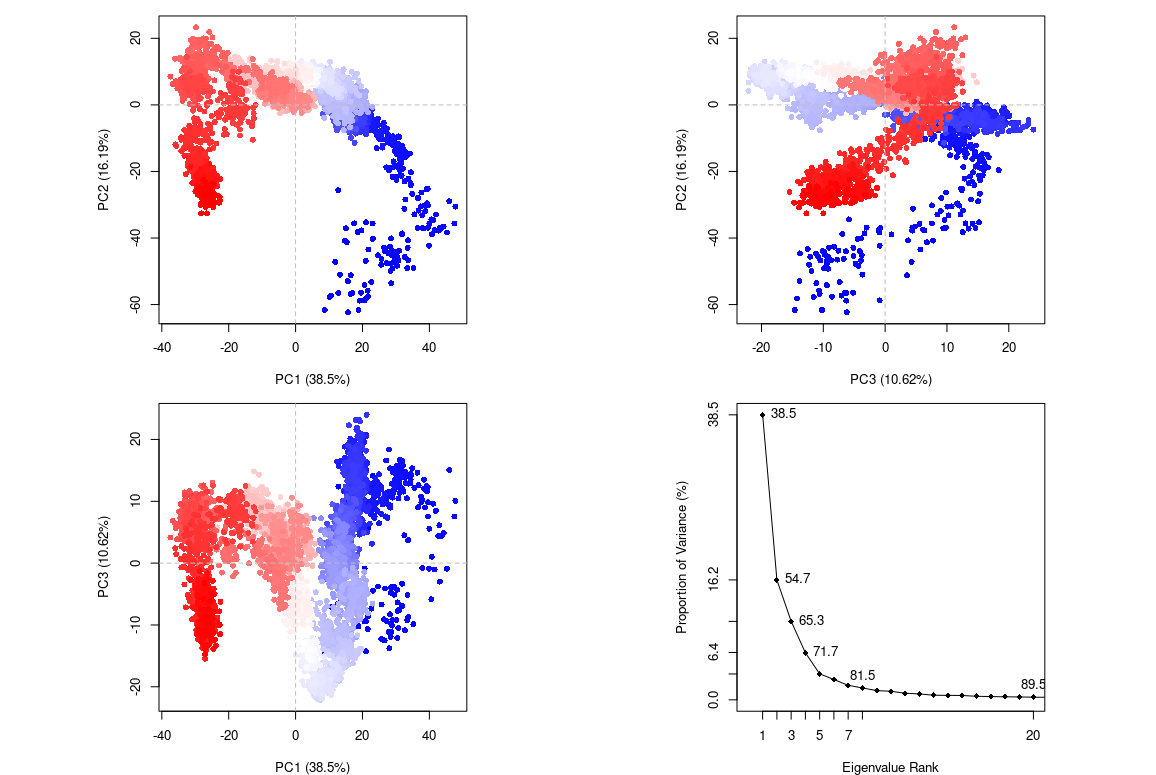

Supplement: Supplementary file 5 [file Image2.TIFF]

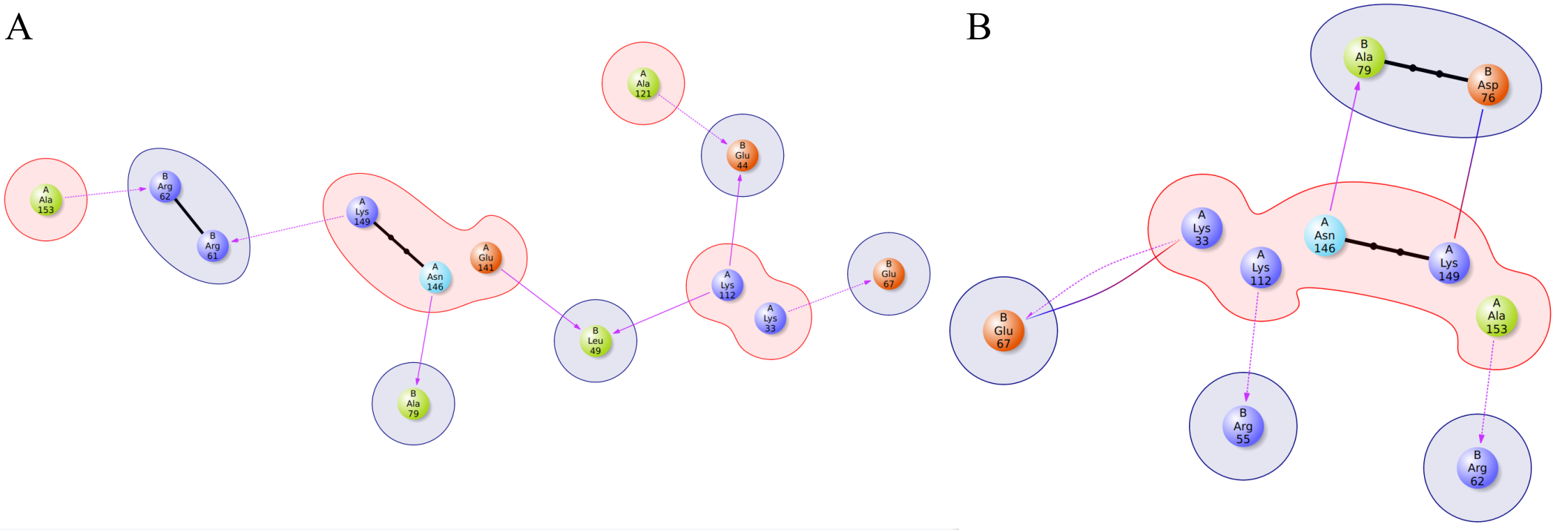

Supplement: Supplementary file 6 [file Image6.PNG]
